# Supplementary material for: Evolution of enhanced innate immune evasion by the SARS-CoV-2 B.1.1.7 UK variant
Source: bioRxiv. 2021 Jun 7:2021.06.06.446826. Preprint. [Version 1] doi: 10.1101/2021.06.06.446826 (PMC8202424; doi:10.1101/2021.06.06.446826)
Supplement: 1 [file NIHPP2021.06.06.446826V1-supplement-1.pdf]

bioRxiv preprint doi: https://doi.org/10.1101/2021.06.06.446826; this version posted June 7, 2021. The copyright holder for this preprint (which was not certified by peer review) is the author/funder, who has granted bioRxiv a license to display the preprint in perpetuity. It is made available under aCC-BY 4.0 International license.

Supplemental Figures

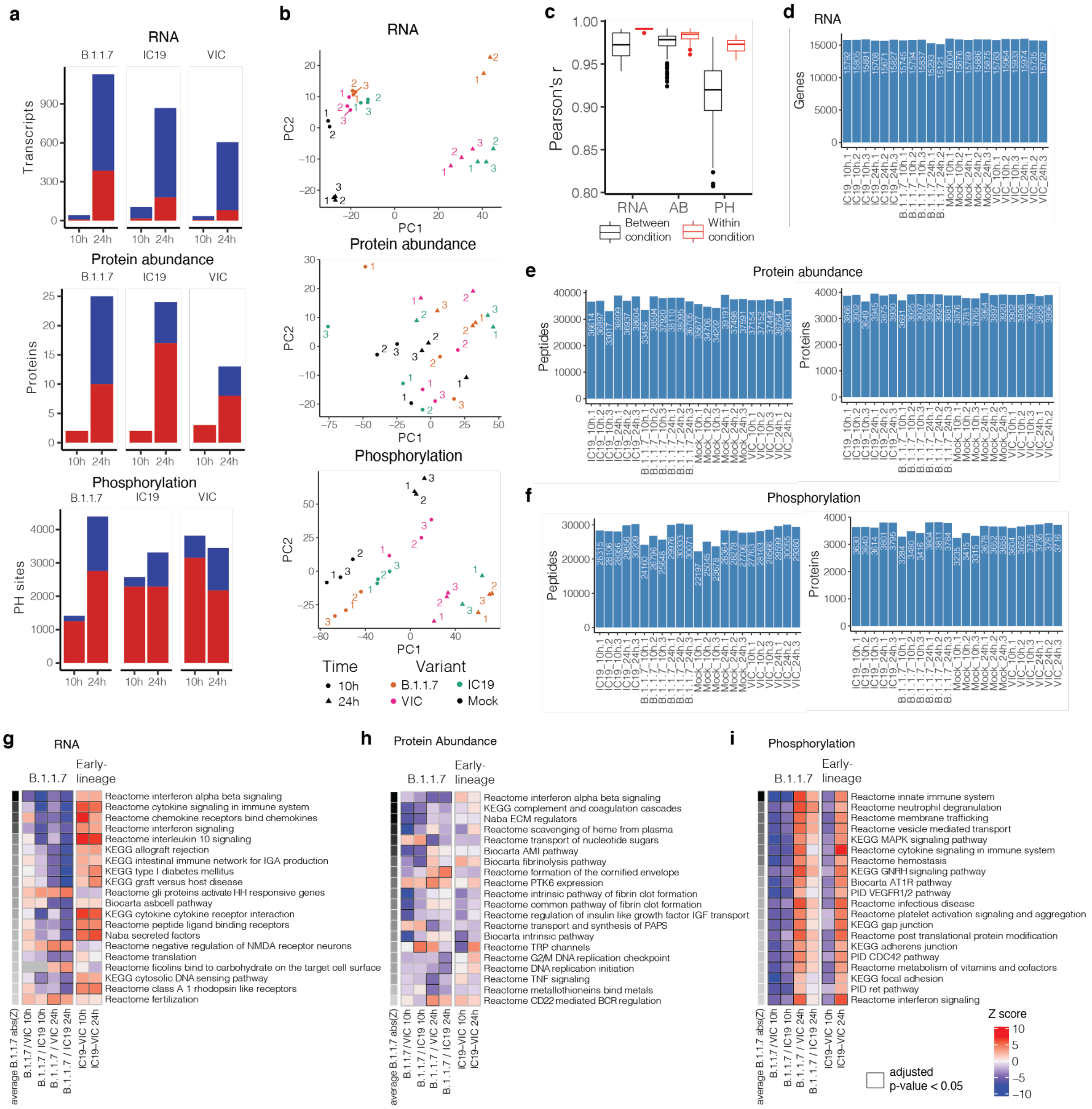

Figure S1. Omics data quality control and pathway enrichments.
a. Significantly changing genes for RNA, proteins for protein abundance, and phosphorylation sites for phosphoproteomics data. Significance was defined as abs(log2FC)>1 and adjusted p-value<0.05. Red depicts positive log2 fold changes whereas blue depicts negative log2 fold changes.
b. Principal components analysis (PCA) on normalised RNA transcripts per million (TPM), protein intensities, or phosphorylation site intensities. Non-finite values were removed and detections (transcripts, proteins, or phosphorylation sites) not shared (non-finite) between all conditions were discarded prior to analysis.
c. Pairwise Pearson's correlation between RNA, protein, or phosphorylation site abundance among replicates within the same condition (red) or between distinct conditions (black).
d. Number of genes expressed above baseline in RNAseq dataset per replicate.

bioRxiv preprint doi: https://doi.org/10.1101/2021.06.06.446826; this version posted June 7, 2021. The copyright holder for this preprint (which was not certified by peer review) is the author/funder, who has granted bioRxiv a license to display the preprint in perpetuity. It is made available under aCC-BY 4.0 International license.

e. Number of peptides and proteins detected per replicate in the abundance proteomics dataset.
f. Number of phosphorylated peptides and corresponding proteins from phosphoproteomics dataset.
g. Gene set enrichment analysis based on log2FC method using RNA dataset (as in Fig. 2b, see Methods). Ranking is based on the average of the absolute value z-scores across the indicated contrasts involving B.1.1.7 (per row). Enrichments with an adjusted p-value<0.05 are indicated with a black border.
h. Same as in g, but for abundance proteomics dataset.
i. Same as in g, but for phosphoproteomics dataset. If a protein possessed multiple phosphorylation sites, the maximum absolute value log2FC was used as the representative value for the protein. Finite values (non-infinite) were prioritised over quantitative values.

bioRxiv preprint doi: https://doi.org/10.1101/2021.06.06.446826; this version posted June 7, 2021. The copyright holder for this preprint (which was not certified by peer review) is the author/funder, who has granted bioRxiv a license to display the preprint in perpetuity. It is made available under aCC-BY 4.0 International license.

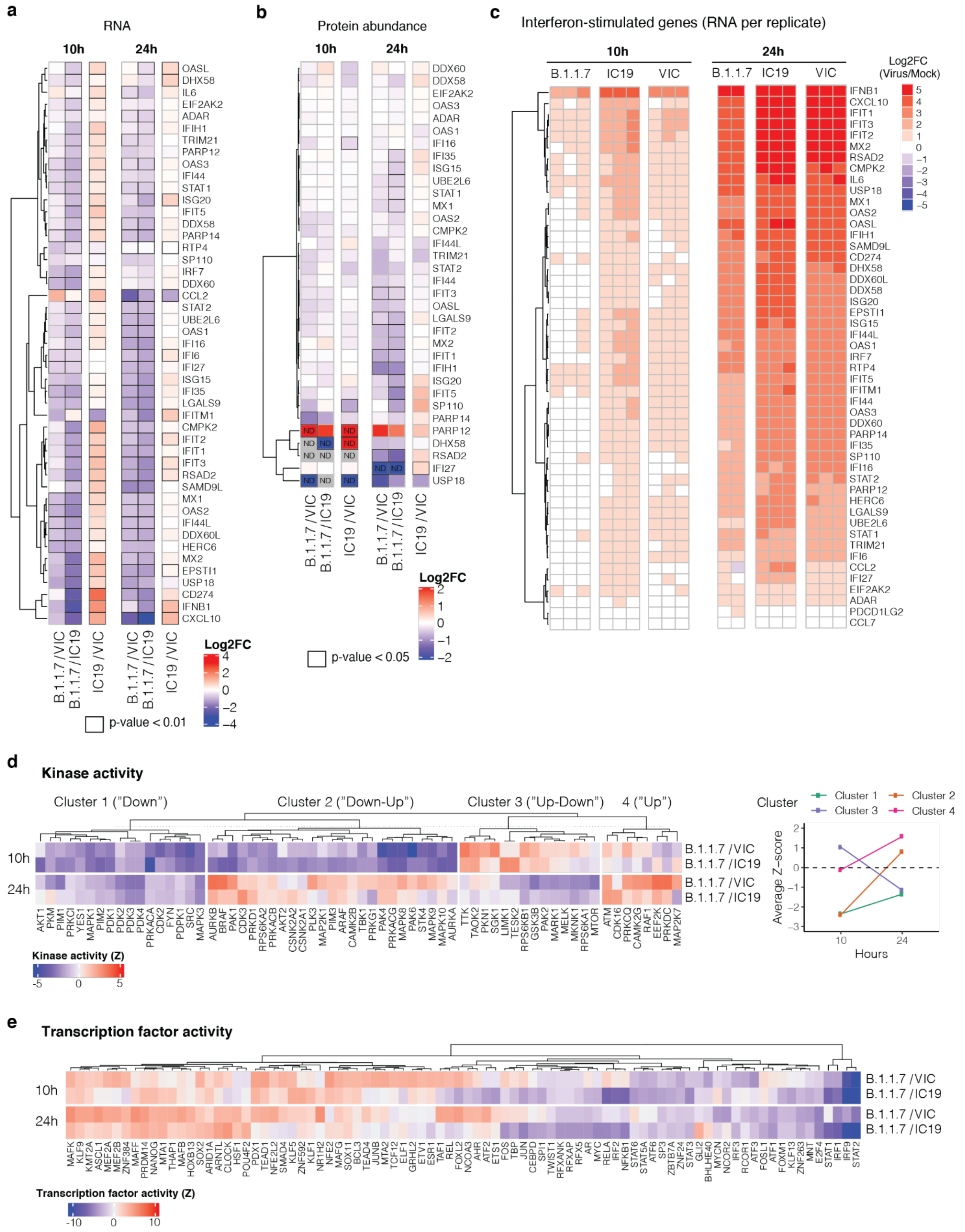

Figure S2. Omics data highlights recruitment of innate immune signaling.
a. Expression of interferon-stimulated genes from Lui et al (2018)[36] (see Methods) using the RNAseq

bioRxiv preprint doi: https://doi.org/10.1101/2021.06.06.446826; this version posted June 7, 2021. The copyright holder for this preprint (which was not certified by peer review) is the author/funder, who has granted bioRxiv a license to display the preprint in perpetuity. It is made available under aCC-BY 4.0 International license.

dataset. Significant fold changes with an adjusted p-value<0.05 are indicated with black borders.
b. Same as in (a) using the abundance proteomics dataset. N.D. indicates proteins either not detected in one condition (thus, Inf or -Inf) or not detected in both conditions.
c. RNA expression per biological replicate of interferon-stimulated genes (ISGs) for each virus versus mock.
d. Full kinase activity analysis of indicated contrasts. Only kinases with an absolute value z-score>2 were kept. Kinases were separated into four distinct clusters using k-means clustering, which naturally reveals groups depicting kinases downregulated for the entire time course ("Down"), downregulated early and upregulated late ("Down-Up"), upregulated early and downregulated late ("Up-Down"), or upregulated or constant throughout the time course ("Up"). Panel on right depicts the average Z-score for each distinct cluster per time point, collapsing across B.1.1.7/VIC and B.1.1.7/IC19 comparisons.
e. Transcription factor (TF) activities were estimated from the RNAseq dataset using known TF-target gene interactions (see Methods). Only transcription factors with an absolute value NES>2.5 were kept. TF are clustered using ward hierarchical clustering based on similar activity patterns across time.

bioRxiv preprint doi: https://doi.org/10.1101/2021.06.06.446826; this version posted June 7, 2021. The copyright holder for this preprint (which was not certified by peer review) is the author/funder, who has granted bioRxiv a license to display the preprint in perpetuity. It is made available under aCC-BY 4.0 International license.

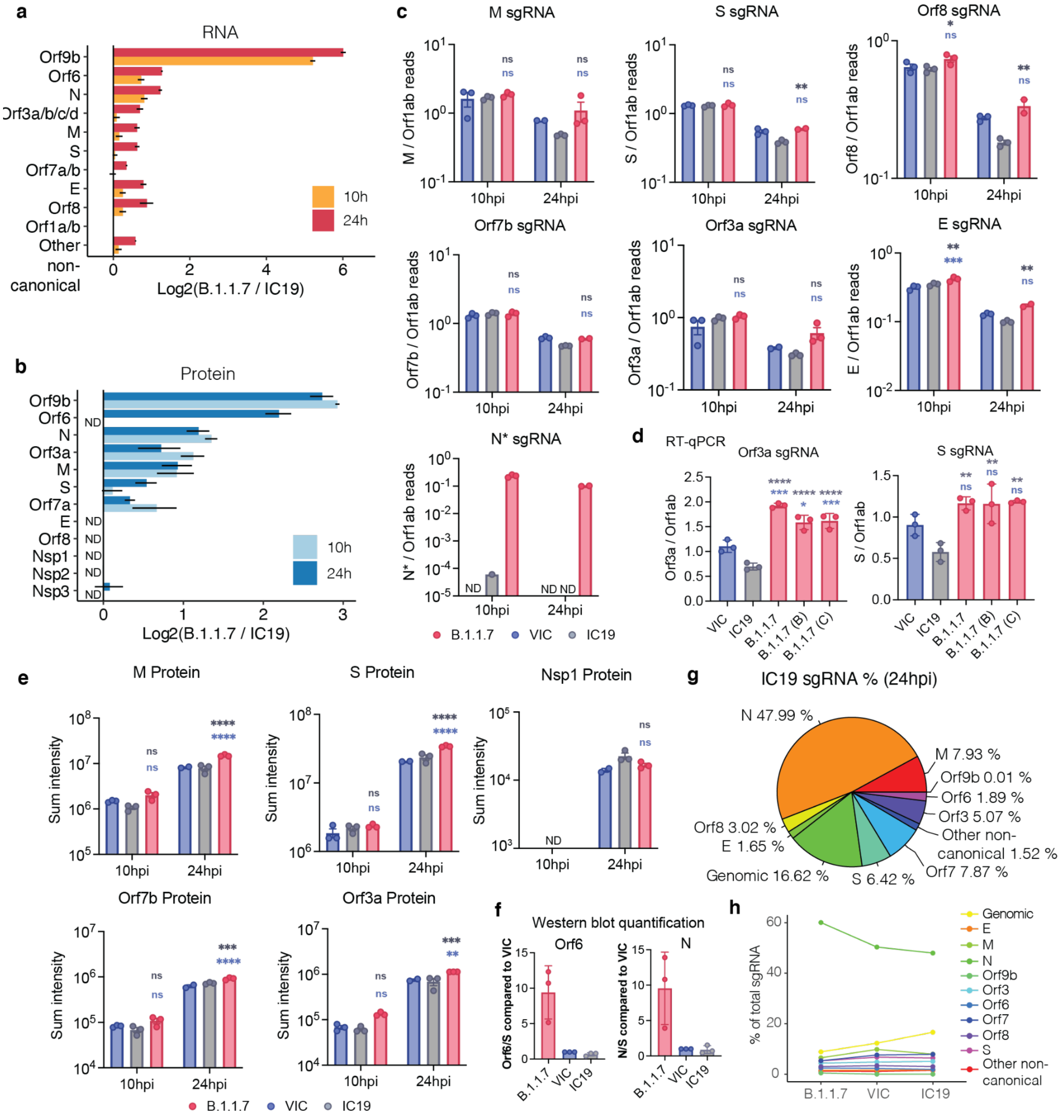

Figure S3. Expression of viral RNA and protein for SARS-CoV-2 variants.
a. Log2 ratio of B.1.1.7 to IC19 subgenomic RNA (sgRNA) abundance as determined from the RNAseq dataset. sgRNA reads are counted only if they possess a leader sequence and normalised to total genomic RNA per time point and virus (see Methods).
b. Log2 ratio of B.1.1.7 to IC19 viral proteins quantified as determined from the abundance proteomics dataset. Peptide intensities are summed per viral protein. Only peptides detected in both B.1.1.7 and IC19 are used for quantification. Bars depict the mean of three biological replicates. Error bars depict the standard error.
c. Quantification of sgRNAs for M, S, Orf8, Orf7a, Orf3a, E and N* from the RNAseq dataset. Counts are normalised to genomic RNA abundance at each time point and virus.

bioRxiv preprint doi: https://doi.org/10.1101/2021.06.06.446826; this version posted June 7, 2021. The copyright holder for this preprint (which was not certified by peer review) is the author/funder, who has granted bioRxiv a license to display the preprint in perpetuity. It is made available under aCC-BY 4.0 International license.

d. Quantification of Orf3a (left) or S (right) sgRNA abundance via RT-qPCR in distinct B.1.1.7 isolates, VIC, or IC19.
e. Summed peptides per viral protein for M, S, Nsp1, Orf7b, and Orf3b from the abundance proteomics dataset.
f. Quantification of Orf6 and N protein from western blot in Figure 3f for B.1.1.7, VIC, and IC19.
g. Pie chart depicting proportion of total sgRNA mapping to each viral sgRNA (containing leader sequence) for IC19.
h. Comparison of percentages of total sgRNA mapping to each viral sgRNA across B.1.1.7, VIC, and IC19.
* (p<0.05), ** (p<0.01), *** (p<0.001), **** (p<0.0001). ns: non-significant.

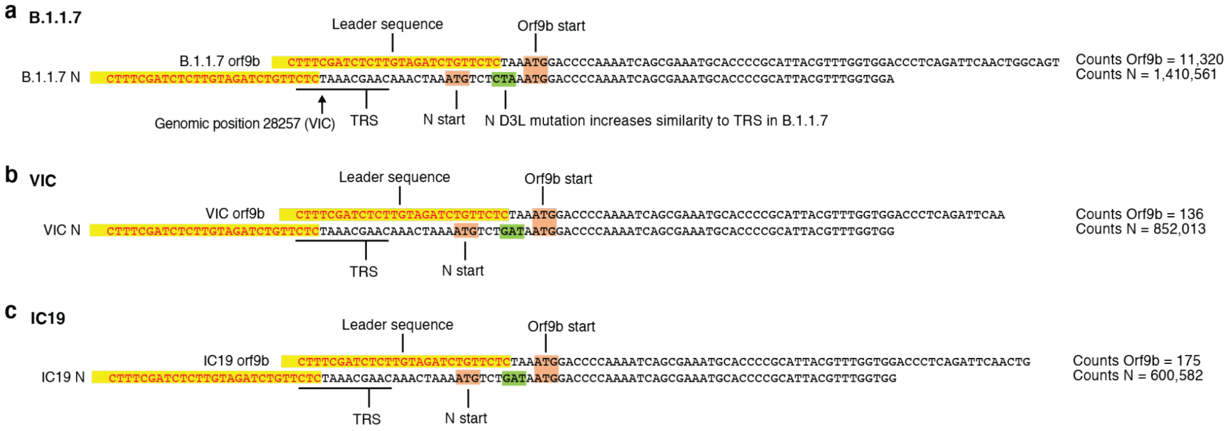

Figure S4. Examples of leader-containing reads for Orf9b and N from RNAseq dataset.
a. Representative sequence for Orf9b (top) and N (bottom) sgRNA from B.1.1.7. Leader sequences used in this analysis to identify sgRNAs are highlighted in yellow. The sequence following the leader sequence is used to differentiate Orf9b versus N sgRNAs. Orf9b and N start codons are indicated in maroon. The site of the N-protein D3L mutation is indicated in green, which results in increased similarity to the transcriptional regulatory sequence (TRS) for B.1.1.7. Read counts of Orf9b and N are indicated to the right. Counts are normalized to mean genomic reads per replicate.
b. Same as in a but for VIC.
c. Same as in a but for IC19.
